# Underlying Processes of an Inverted Personalization Effect in Multimedia Learning – An Eye-Tracking Study

**DOI:** 10.3389/fpsyg.2017.02202

**Published:** 2017-12-15

**Authors:** Steffi Zander, Stefanie Wetzel, Tim Kühl, Sven Bertel

**Affiliations:** ^1^Instructional Design, Faculty of Art and Design, Bauhaus-Universität Weimar, Weimar, Germany; ^2^Usability Research Group, Faculty of Information and Communication, Flensburg University of Applied Sciences, Flensburg, Germany; ^3^Psychology of Education, Faculty of Social Sciences, University of Mannheim, Mannheim, Germany

**Keywords:** multimedia learning, personalization effect, eye-tracking, cognitive load, multimedia design principles

## Abstract

One of the frequently examined design principles in multimedia learning is the personalization principle. Based on empirical evidence this principle states that using personalized messages in multimedia learning is more beneficial than using formal language (e.g., using ‘you’ instead of ‘the’). Although there is evidence that these slight changes in regard to the language style affect learning, motivation and the perceived cognitive load, it remains unclear, (1) whether the positive effects of personalized language can be transferred to all kinds of content of learning materials (e.g., specific potentially aversive health issues) and (2) which are the underlying processes (e.g., attention allocation) of the personalization effect. German university students (*N* = 37) learned symptoms and causes of cerebral hemorrhages either with a formal or a personalized version of the learning material. Analysis revealed comparable results to the few existing previous studies, indicating an inverted personalization effect for potentially aversive learning material. This effect was specifically revealed in regard to decreased average fixation duration and the number of fixations exclusively on the images in the personalized compared to the formal version. These results can be seen as indicators for an inverted effect of personalization on the level of visual attention.

## Introduction

Imagine you are learning facts about brain hemorrhage and the learning material contains sentences like:

“*When one of your vessels is hurt and blood cannot leak from your brain” or “Parts of your brain can be destroyed as a consequence of the enormous high pressure*.”

Would you continue reading? Several studies from the field of multimedia found evidence that a personalized language style has a positive effect on motivation and learning outcomes of learners (e.g., Mayer, 2014). This positive effect is known as personalization principle. According to the Cognitive Load Theory (CLT; Chandler and Sweller, 1991; Paas et al., 2003; Sweller, 2005), optimal computer-based learning material induces as low a cognitive load (CL) as possible to enable learners to use their free cognitive resources to process important information (Mayer and Moreno, 2003; Van Merrienboer et al., 2003; Sweller, 2005). A reduction of CL can be achieved through different design principles that aim in supporting perception and processing of information, as for instance the contiguity or modality principle (Mayer, 2014). Moreover, it is assumed that greater interest in the learning material results in a more effective use of available cognitive resources, as interest frees up mental resources (e.g., attention, persistence) of learners and supports focused allocation of these resources to the domain of interest (Hidi et al., 1992; Harp and Mayer, 1998). In turn, the higher availability of resources combined by focused attention should reduce cognitive load while processing learning materials in high interested compared to low interested learners. The personalization of the language style is seen to be one approach to enhance the situational interest of learners, and hence to reduce CL.

In recent research, the personalization effect was investigated within a wide, but mainly neutral, non-aversive range of domains and learning materials, such as lightning (Moreno and Mayer, 2000), botany (Moreno and Mayer, 2000, 2004), astrophysics (Kartal, 2010), computer technology (Rey and Steib, 2013), psychology (Reichelt et al., 2014), and anatomy (Mayer et al., 2004; Ginns and Fraser, 2010; Stiller and Jedlicka, 2010; Schworm and Stiller, 2012). However, the effect of personalization on aversive learning content has not been researched yet, although the assumption that the specific topic of the learning material might influence the beneficial effects of addressing learners personally has been mentioned before (Ginns et al., 2013; Reichelt et al., 2014). As one can derive from the two example sentences at the beginning of the section, the use of a personalized language style in aversive learning content (e.g., brain hemorrhage) might cause different or even inverted effects, compared to neutral learning content (e.g., botany). First evidence of an inverted effect was reported by Kühl and Zander (2017). The authors found that learning outcome and cognitive load were negatively affected by personalized messages in a learning material on causes and symptoms of cerebral hemorrhage. They argued that learning material of an aversive and threatening character would result in some kind of avoiding behavior, particularly when learners receive the personalized material. However, data to support this argumentation as well as systematic research on this issue does not exist and moreover, the underlying processes of such an inverted effect remain unclear.

The aim of the present study is to examine how such an inverted effect of personalized language styles for potentially aversive learning content can be explained. In order to analyze potential explanatory factors including cognitive load, learning time and state anxiety, the eye movements of the learners were analyzed during the learning phase, based on the hypothesis that the personalization of the aversive learning content would cause differences in the direction and focus of attention, and therefore in gaze behavior.

## Explanations of the Personalization Principle

The personalization principle can be used to promote learning with multimedia resources by replacing impersonal articles with possessive pronouns and by directly addressing learners (i.e., using the second person). The effect behind the personalization principle can be discussed from at least two perspectives, namely (1) by self-reference and (2) based on social cues.

The self-referential effect (Rogers et al., 1977; Klein and Loftus, 1988; Symons and Johnson, 1997) is one theoretical explanation of the personalization principle. According to this effect, information can be better organized and incorporated when it is related to the self. This relation of information to the learners themselves results in a better processing of the learning content (Craik and Lockhart, 1972; Rogers et al., 1977; Moreno and Mayer, 2000; Reichelt et al., 2014) and can result in better learning outcomes (Mayer, 2009).

According to the Social Agency Theory (SAT) (Mayer, 2009), personalized learning material contains social cues. It is assumed that instructions enriched by social cues trigger a social response in the learner. In this context, a social response is the learner’s feeling of stronger social bonds to the instructor (Moreno and Mayer, 2004). Thus, the learners feel like they are in a social interaction (e.g., with a pedagogical agent; see also Reeves and Nass, 1996). It is assumed that learners are willing to invest more effort when they have the feeling that they are interacting socially with the computer (see also Reichelt et al., 2014). In turn, this response can improve the active cognitive processing, resulting in better comprehension of the learning material (Hidi and Baird, 1988; Hidi et al., 1992). Active cognitive processing refers to the attention of the learner, which is guided to relevant parts of the learning material (Moreno and Mayer, 2004). The acquired knowledge will then be organized in coherent mental representations, which will in turn be integrated with the learner’s prior knowledge.

### Personalization in Aversive Learning Material

In addition to areas of learning that can be considered as neutral or non-aversive, such as topics from the fields of physics or engineering, there are also topics that a lot of people consider as emotionally aversive or emotionally loaded. This is the case for lethal and suddenly occurring diseases (e.g., Kühl and Zander, 2017) or topics like child labor (Reichelt, 2015). Emotionally aversive topics, like health issues, might be related to a heightened negative emotional state (e.g., state anxiety; cf. Laux et al., 1981). Learners may not want to engage deeply with such aversive content, since the connection to their life is perceived as threatening. Hence, it is not likely that learners want to relate such content even closer to themselves or to perceive a stronger social relation to the issue (through either self-reference or a social response). Personalized material with aversive content might thus cause learner avoidance of any deeper processing of the material. In turn, this would result in detrimental effects on mental effort (e.g., cognitive load) and knowledge acquisition. In contrast, using formal language when dealing with potentially aversive content would likely make learners feel more comfortable with the material and thus stop them from avoiding the active processing of information that results in a better understanding (Kühl and Zander, 2017).

It should be noted that these assumptions can also be brought in line with the social agency explanation of the personalization effects. As mentioned in the section above, the SAT postulates that personalization increases the dialog-orientation with the computer, involvement and learning outcomes. However, if the learner is confronted with an “awkward topic” by the computer in a very dialog-oriented way, the learner will likely feel uncomfortable. At this, the social response fails to show the beneficial effect of personalized messages and blocks greater involvement, active cognitive processing and, as a result, a better understanding. In contrast, the social response triggered by an inappropriate language style might lead to avoiding behavior instead of deeper involvement with the learning material. As a consequence, for aversive learning content, a formal, more distanced language style might show a more positive learning effect compared to a personalized, more dialog-oriented language style.

The first evidence for an inverted effect of a personalized language style in the framework of potentially aversive material was reported by Kühl and Zander (2017). In two studies, learning material on the symptoms of cerebral hemorrhages was presented either in a formal or personalized version. Results of both studies showed the expected reversed effect regarding transfer. As underlying processes the authors assumed firstly that the inverted effect could be traced back to a higher state anxiety accompanied by a greater cognitive load in the personalized version of the material. In this regard personalized messages in potentially aversive learning material (e.g., causes and symptoms of cerebral hemorrhages) were seen to increase state – anxiety and subsequently to increase the load on the limited capacity of working memory (Eysenck, 2013). However, the assumptions on these underlying processes were not confirmed consistently. The state anxiety increased in the personalized as well as in the formal version in both experiments can therefore cautiously be interpreted as an indicator that the material is indeed of an aversive nature. The cognitive load was higher in the personalized version only in experiment 2, but did not differ between language styles in experiment 1. As a consequence of the lack of explanation by means of cognitive load and state anxiety in experiment 1, learning time was measured as a temporal indicator for the assumed avoidance behavior in experiment 2. However, learning time did not differ either way. Hence, the underlying processes of an inverted effect remain unclear.

Most of the measurements applied in studies on personalization are mainly based on self-reporting and therefore on subjective measures (Ginns et al., 2013). The application of psychophysical methods, such as eye-tracking, EEG or galvanic skin response, is preferable (Ginns et al., 2013) and potentially useful to examine the underlying cognitive processes during learning with multimedia content. Indeed, Zander et al. (2015) have already shown that eye movements are an indicator for the depth and/ or direction of information processing in multimedia learning under different personalization conditions.

The study was conducted with neutral, non-aversive learning material on weather phenomena that was presented either in a personalized or formal style. As most important parameters reading depth and transition count were inspected. Reading depth is defined as the accumulated time spent looking at the areas of interest (AOI) divided by the area in cm^2^. This measure indicates how much of the text is read or how much of a picture has been examined. The AOIs refer to those areas with registered eye movements on the screen which are defined to be relevant to be inspected by the researcher. Transition count was seen as an indicator for a better mental integration of the presented textual and pictorial information (Holsanova et al., 2009; Johnson and Mayer, 2012).

Data on gaze behavior for the neutral material revealed that learners in the personalized condition did not show greater reading depth in general (text and picture AOIs combined), but rather exclusively for the picture AOIs. Moreover, the number of transitions between text and picture AOIs was greater for the personalized learning material. Both results indicate that learners inspect more of the pictorial information and further show a better mental integration of information sources (e.g., textual and pictorial) in the personalized compared to the formal language style version. However, although these findings are promising, the expected corresponding higher learning outcomes and reduced cognitive load in the personalized condition were revealed by trend, but not on a statistically significant level.

## Research Questions and Hypotheses

The presented data and theoretical background show that two of the open questions regarding the personalisation principle are (1) whether the inverted effect of personalized language style for aversive material that was found in previous studies can be replicated and (2) if so, whether it can be explained by an inverted pattern of gaze behavior as compared to neutral, non-aversive learning material.

As the literature review showed, the effect of the personalization principle on attention processes has been neglected so far (Ginns et al., 2013; Zander et al., 2015). Nevertheless, the above-mentioned first studies have shown that eye-tracking constitutes an appropriate objective, process-oriented measure to examine differences in the allocation of attention resources. Analogous to the reported findings, we based our hypotheses on the eye-tracking parameters according to the study by Zander et al. (2015). We expected the opposite effects regarding gaze behavior and the corresponding eye-tracking parameters. With respect to cognitive load and learning outcomes, our assumptions correspond to those by Kühl and Zander (2017) in order to replicate the inverted personalization effect. Based on current research we derive the following hypotheses:

Hypothesis 1.1: Learners who receive a formal version of potentially aversive computer-based learning material show superior performance on retention and transfer compared to those who receive a personalized version.
Hypothesis 1.2: Learners who receive a formal version of a potentially aversive learning material report lower cognitive load compared to those receiving a personalized version.
Hypothesis 2.1: Learners who receive a formal aversive computer-based program show higher values for fixation rate and fixation duration and the calculated reading depth compared to those who receive a personalized version.
Hypothesis 2.2: Learners who receive a formal aversive computer-based program have more transitions between text and image areas compared to those who receive a personalized version.

In order to investigate the research questions and hypotheses presented above, we conducted the following experiment.

## Eye-Tracking Study: Methods and Material

### Participants and Design

The participants were 37 university students from the Bauhaus-Universität Weimar, Germany and University of Erfurt, Germany (mean age = 25.1, *SD* = 3.92, male = 19). Learners were tested in single sessions in the Usability Lab of the Bauhaus-Universität Weimar and were randomly assigned either to a personalized (*n* = 19) or to a formal (*n* = 18) version of a computer-based program about brain hemorrhage. Randomisation was realized by order: starting with a participant in the formal version, the next participant was presented with the other of the two versions (e.g., personalized or formal) version. Although ethical approval in Germany is not specifically required by a committee (Hearnshaw, 2004), researchers in Germany are bound to the Declaration of Helsinki. At this, to assure that the interests of the participants were protected, in our study every participant was first informed about aims, content, expected benefit, potential risks, and methodology of the study. They were also informed that participation in the study can be declined and that the given consent can be withdrawn at any time without any consequences. After informing, participants signed the voluntary consent and gave written informed consent in accordance with the Declaration of Helsinki.

### Learning Material

The presented learning material consisted of static diagrammatic illustrations and text passages that were placed side by side. The basic structure was equal to the material used by Kühl and Zander (2017), but pictures and text were adapted regarding dissolution of the pictures and text length per screen. The learning phase was self-paced and had a total duration of approximately 7 min per participant. Following the recommendations of Mayer (2014), we used the following technique for creating a personalized style of the text as can be seen in **Table 1**. The formal text was personalized by replacing impersonal articles with possessive pronouns and third-person constructions with second-person constructions. The material consisted of six slides explaining the anatomy of the skull with the brain and the brain skins in the short introductory part. This was followed by causes and symptoms of cerebral hemorrhages in the main part.

**Table 1:** Examples of personalized and formal text versions.

Formal style	Personalized style
*The* task is…	*Your* task is…
The hemorrhage above *the* dura mater*…*	The hemorrhage above *your* dura mater …

### Procedure and Measures

As a first step, prior experiences of the learners regarding brain hemorrhages in their family were assessed by asking them to answer with yes or no. In the case of “yes” participant were asked to take part in another study This was the case with none of the participants. After this, the participants were placed 55–60 cm from a 24-inch monitor. An SR Research EyeLink II eye tracker was adjusted and calibrated using a nine-point calibration. Fixations, saccades and blinks were recorded at 250 Hz for the dominant eye of each participant during the learning phase. They received either a personalized or formal learning material. (1) After completing the learning phase, participants rated how inviting and personally appealing they found the language style based on two attributes (“personal” or “formal” on a five-point-Likert scale ranging from 1- does not apply at all to 5- applies completely). This was done as a manipulation check. (2) We furthermore measured the perceived cognitive load as a measure of perceived difficulty (Koch et al., 2008), These ratings were provided on a seven-point-Likert scale, ranging from 1- extremely easy to 7- extremely difficult (3) As additional measures, we recorded the state anxiety (Laux, 1981) and learning time in accordance with previous experiments on the inverted personalization effect. (4) Moreover, positive and negative affect were recorded using the PANAS questionnaire (Watson et al., 1988; Krohne et al., 1996) to control for more fine-grained changes in affect, as previous studies found that the state anxiety did not explain the inverted personalization effect. The PANAS consists of two 10-item mood scales and measures positive affect (e.g., enthusiastic, attentive, inspired) and negative affect (e.g., afraid, upset, distressed). Participants rate the extent to which they have experience the emotions in the actual situation on a five-point scale ranging from 1- very slightly or not at all, 2- a little, 3- moderately, 4- quite a bit to 5- very much.

(5) Following this, participants gave responses on the retention and a transfer test (to check learning outcomes).

### Data Analyses

**FIGURE 1:**
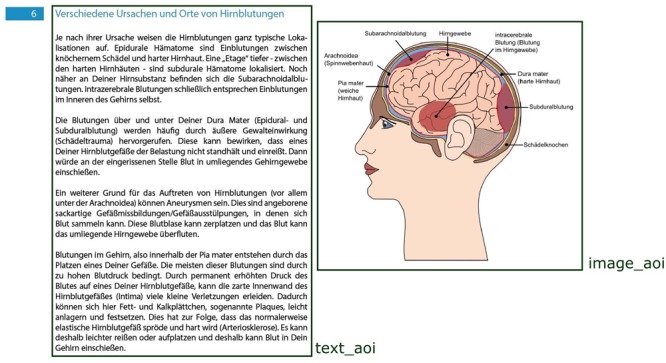
Predefined AOIs based on hypotheses.

The gaze data was analyzed based on the average fixation duration, the number of fixations, the reading depth and the transitions between certain areas. Based on our hypotheses regarding the personalization effect and a cluster analysis of the gaze data, the screen was divided into *AOIs* (see **Figure 1**).

Numbers of fixations, average fixation duration and reading depth were analyzed using the hypotheses-based AOIs, since they had an equal size in both conditions and less than 5% of fixations were found to lie outside of these AOIs. Overall, two main AOIs were defined, one containing the text (*text_AOI*) and the other containing the image (*image_AOI*). Additionally, the number of fixation transitions between pairs of AOIs (i.e., a fixation on one AOI followed by a fixation on another AOI, irrespective of transition direction) were considered.

## Results

Multivariate analysis of variance (MANOVA) was applied for the gaze-based dependent variables. The data from the questionnaires and the learning outcomes were analyzed using one-way ANOVAs. Effect sizes are reported as partial Eta squared ($\eta_{p}^{2}$) for the MANOVA and d for ANOVAs [interpreted after (Cohen, 1988) with 0.2 as small, 0.5 as medium and 0.8 as large effect for Cohen’s d].

### Manipulation Check

The manipulation check revealed significantly higher values for the personal appeal of the language in the personalized condition and significantly higher values for the formal appeal in the formal condition (see **Table 2** for mean values), indicating that the manipulation of language style indeed led to differences in the perception of the language style appeal.

**Table 2:** Means and standard deviations for questionnaire data and learning outcomes for formal and personalized conditions (Hypotheses 1.1 and 1.2).

Variables and measures	Formal (*N* = 18)		Personalized (*N* = 19)		
	Mean	*SD*	Mean	*SD*	
**Manipulation check**					
Perceived personal appeal of language style	1.72	0.83	2.63	1.47	*F*(1,35) = 5.347, *p* = 0.027, *d* = 0.77
Perceived formal appeal of language style	4.11	0.76	3.58	0.77	*F*(1,35) = 4.489, *p* = 0.041, *d* = 0.72
**Learning outcome and CL**					
Retention	7.28	2.11	7.11	1.99	*F*(1,35) = 0.065, *p* = 0.80, *d* = 0.08
Transfer	8.28	2.42	7.32	2.49	*F*(1,35) = 1.414, *p* = 0.242, *d* = 0.39
Cognitive load	4.06	1.39	4.17	1.15	*F*(1,35) = 0.068, *p* = 0.795, *d* = 0.088
**Additional measures**					
PANAS positive	2.68	0.75	2.59	0.57	*F*(1,35) = 0.172, *p* = 0.681, *d* = 0.14
PANAS negative	1.32	0.35	1.29	0.32	*F*(1,35) = 0.118, *p* = 0.733, *d* = 0.11
State anxiety	41.25	4.84	40.24	4.28	*F*(1,35) = 0.408, *p* = 0.527, *d* = 0.22
Learning time in minutes	7.26	1.82	6.92	1.63	*F*(1,35) = 0.409, *p* = 0.527, *d* = 0.21

### Hypotheses 1.1 and 1.2: Perceived Language Style Characteristics, Cognitive Load, and Learning Outcomes

**Table 2** provides an overview of means and standard deviations for the perception of the language style, cognitive load, state anxiety, positive/negative affect, learning outcomes, and the learning time.

As far as learning outcomes are concerned, no differences were found for retention. For transfer, learners who received the formal text achieved somewhat higher values compared to the personalized version. However, these differences failed to reach statistical significance. For cognitive load, there was no difference between the conditions. Hypothesis 1.2 was therefore not confirmed. Additionally, measurements for state anxiety and for the positive and negative affect from the PANAS questionnaire, did not reveal significant differences between conditions. This measure therefore does not reveal any more fine-grained results with respect to possible affective variables that could explain the inversion of the effect. The measurement of learning time also revealed no significant differences, respectively.

### Hypotheses 2.1 and 2.2: Eye-Tracking Analysis

**Table 3** shows the means for gaze-based variables that were included in the MANOVA for both personalized and formal presentation styles. The MANOVA revealed a significant multivariate effect between the conditions [*V* = 0.384, *F*(7,29) = 2.578, *p* = 0.034, $\eta_{p}^{2}$ = 0.384].

**Table 3:** Means, standard deviations and between-subject effects for gaze data variables for both conditions (Hypothesis 2.1 and 2.2).

Gaze data parameters	Formal (*N* = 18)		Personalized (*N* = 19)		
	Mean	*SD*	Mean	*SD*	
Average fixation duration on text_AOIs (ms)	168.64	22.56	187.22	16.54	*F*(1,35) = 8.22, *p* = 0.007, *d* = 0.95
Number of fixations on text_AOIs (%)	76.88	2.72	81.19	3.40	*F*(1,35) = 17.937, *p* < 0.001, *d* = 1.39
Reading depth on text_AOIs (s/cm^2^)	171.01	42.06	177.72	39.97	*F*(1,35) = 0.248, *p* = 0.621, *d* = 0.16
Average fixation duration on image_AOIs (ms)	80.87	13.50	66.32	16.88	*F*(1,35) = 8.323, *p* = 0.007, *d* = 0.98
Number of fixations on image_AOIs (%)	30.95	4.07	24.70	5.51	*F*(1,35) = 15.223, *p* < 0.001, *d* = 1.32
Reading depth on image_AOIs (s/cm^2^)	91.19	38.66	69.05	28.39	*F*(1,35) = 3.972, *p* = 0.054, *d* = 0.68
Transitions between text and picture AOIs (#transitions/s)	0.18	0.06	0.14	0.05	*F*(1,35) = 3.646, *p* = 0.064, *d* = 0.63

Subsequent analyses for the gaze-based parameters revealed, significant univariate effects depending on the AOIs, for either text or image.

#### Text_AOIs

The average fixation duration on the *text_AOIs* was significantly higher in the personalized condition. The same applies to the number of fixations on the *text_AOIs*, which was significantly higher in the personalized condition. In contrast, the reading depth of the *text_AOIs* was very similar in both conditions and revealed no significant difference. Hypothesis 2.1 was thus not confirmed for the *text_AOIs*. Contrary to expectations, results related to fixation duration and number of fixations on the *text_AOIs* were higher in the personalized compared to the formal language condition.

#### Image_AOIs

Average fixation duration on the *image_AOIs* and the number of fixations on the *image_AOIs* were significantly higher in the formal condition. According to Rayner and Pollatsek (1988), a greater average fixation duration indicates more effortful cognitive processing. These values thus indicate an aversive gaze behavior in the personalized condition: one that is restricted to the *image_AOIs*. This interpretation is also supported by the values for reading depth on images which point into the same direction. The present lower values relating to the *image_AOIs* in the personalized condition suggest less intense observation of the images compared to the formal condition. However, the results for reading depth – which indicates how much of the text is read or how much of a picture is examined (Holmqvist et al., 2012) – are not significant. Hypothesis 2.1 could therefore be confirmed with regard to the variables, fixation rate and average fixation duration. However, this applies only to the pictorial information.

#### Transitions between Text_AOIs and Image_AOIs

The number of transitions between *text_AOIs* and *image_AOIs* is descriptively lower for the personalized learning material. However, the difference is not significant. A greater number of transitions between AOIs with semantic relations indicates better connection and integration of the presented information (Holsanova et al., 2009). Hypothesis 2.2 was therefore not confirmed on a statistically relevant level.

## Discussion

The present study investigated if the personalization effect shifts into the reverse direction when applied to aversive learning material, and how that could be explained. We analyzed the influence of personalization on learning processes and learning outcome variables as well as learners’ eye-movements during the learning phase. The evidence from the present study can be seen as filling the above-mentioned research gaps in two ways: the study can be seen to provide an answer to the current call to search for boundary conditions for the personalization effect as to, for instance, the kind of learning content or the influence of learner prerequisites. It may further provide an explanation as to why the personalized language style has not proven beneficial for learning in some previous studies (e.g., Ginns et al., 2013). It has to be said that, so far, only two studies (Kühl and Zander, 2017) have examined aversive learning material. On the one hand, we built on this lack of information in our research and tested the assumption that the personalization effect in aversive multimedia learning material would vanish or would even be inverted. On the one hand, this was seen as a chance to replicate former findings. On the other hand, we filled a methodological research gap left by the previous studies on the personalization principle in that we combined learning variables with eye-tracking data analyses.

Specifically, the study was based on previous findings on the inverted personalization effect and aimed to investigate explanatory variables that were supposed to allow for a more fine-grained analysis of attention processes underlying the effect. This was done as prior studies have shown inconclusive results for the assumed explanatory variables state anxiety, cognitive load and learning time. With respect to the results for the explanatory variables (e.g., cognitive load, state anxiety, and positive and negative affect) the present study did not reveal any differences. The inconclusive results on cognitive load and state anxiety from previous studies were replicated and both variables did not shed any further light onto the underlying processes.

Based on the theoretical framework (i.e., self-reference effect and SAT), we assumed that learners who received a personalized aversive learning material would avoid deeper involvement with the personalized aversive learning material and that the beneficial effect of personalized language style would be inverted. As a result this would have been reflected in the learning outcomes and the data on gaze-behavior. For learning outcomes (e.g., transfer), this assumption was not confirmed statistically. Descriptive results showed that learners who received the formal language condition achieved higher values for transfer learning outcomes compared to the personalized condition. This descriptive finding is in accordance with the previous findings of studies on the inverted effect, which showed the inversion for transfer, but not for retention. However, the difference was not significant (probably because of a power problem) and therefore can only be seen as a call to examine this more into deep and with further, larger samples.

The eye-tracking data analysis revealed a significant multivariate difference between both language style conditions, and also significant univariate effects for several gaze-based dependent variables. Nevertheless, the expected combined effects for both, *text-AOIs* and *image_AOIs* combined, and in favor of the formal instead of the personal language style were not found for the inspected gaze parameters (e.g., fixation, fixation duration, and reading depth). Instead, the expected pattern of results was restricted to the *image_AOIs* in the learning material, showing higher fixation duration and number of fixations in the formal compared to the personalized language condition. This indicates more effortful cognitive processing of the material that was presented in the formal language style. Moreover, in regard to the theoretical background of the present study, this value indicates the expected aversive gaze behavior in the inappropriate personalized condition, but only for pictorial information. The effect is further supported by the descriptive data on the gaze parameter *transitions*, which show a trend into the direction that learners in the formal language version show more transitions between *text_AOI*s and *image_AOIs* and therefore more integrative mental processing (Holsanova et al., 2009). Additionally, the descriptive results on greater transfer performance in the formal language condition indicate deeper mental processing and integration of textual and pictorial information in the formal rather than in the personalized condition.

In turn, and as an unexpected result, the average fixation duration and also the number of fixations on the *text_AOIs* were significantly higher in the personalized compared to the formal language condition. These results contradict the hypothesis of avoiding gaze behavior for the *text_AOIs*. Nevertheless, to further interpret and conclude on these findings, they have to be seen in relation to the results on learning outcomes. As was shown, the values for transfer performance point in the direction that learners using the formal language version show a deeper understanding compared to those using the personalized language version. Gaze data for the *text_AOIs* in the personalized version should therefore not necessarily be interpreted as indicators for a deeper processing of the personalized material. To the contrary, taking into account the finding of a descriptively higher transfer performance in the formal compared to the personalized language condition it can be surmised that the longer fixation duration on textual information in the personalized condition can alternatively be traced back to the additional cognitive processing of task-irrelevant thoughts, which do hinder deeper processing of relevant information. A possible explanation in this regard might be that this is a result of annoyance and/or perceived dissonance between information on an uncontrollable, potentially lethal disease and the personalized language style. Future studies should implement interview or thinking aloud techniques to control for these potential reasons.

All in all, although the assumed avoiding gaze behavior was revealed exclusively for images, this is in line with previous findings on gaze behavior in personalized vs. formal language learning material by Zander et al. (2015). For material that was neutral and non-aversive, they found a corresponding pattern of results with an opposite effect exclusively for pictorial but not for textual information. Their data showed higher reading depth only for images, but not for textual information under the personalized condition. At this, the present data give a first insight into the processes underlying the understanding of personalized and formal language in computer based learning materials based on empirical data.

### Limitations and Future Research

As was mentioned before, the sample size and a possible low power can be seen as restricting statistical factors of this study when interpreting the results. Our results seem to accomplish former patterns of results for non-aversive learning material. Nevertheless, when critically reflecting our results one can also conclude that those point into the opposite direction and might tell us that the effects were obtained by chance. Therefore, further research with larger samples including the above described mixed methods would be a fruitful approach.

Regarding the emotional-affective explanatory variables, the assumed reason that personalized aversive learning material reinforced the reported values for state anxiety (Kühl and Zander, 2017) or negative affect could not be confirmed in our study. This is in accordance with former studies by Kühl and Zander (2017) with regards to the state anxiety. Nevertheless, to answer the question of how emotional aspects influence personalization effects in more detail, further studies are needed using multi-dimensional measurements to analyze learners’ emotions in relation to different language styles. A combination of methods like quantitative measurements and qualitative approaches (e.g., semi-structured interviews) can be seen as a good tool for exploring new factors to explain personalization effects and to obtain more detailed information about the participants’ opinions on different language styles.

To analyze the effects more in depth, learner prerequisites should be taken into account more strictly. In the present case, it would be worthwhile to collect data on the area in which the given field participants were studying or working, to examine the personalization effect in relation to their background. Our participants had no medical background, but they differed within their field of studies, ranging from engineering to design and art education. Anecdotal evidence showed that especially students of computer science and engineering perceived the personalized language style as inadequate and childish, so that the results in terms of their affect and emotion might be confounded by factors due more to annoyance. Another learner prerequisite of importance might be how sensitive participants are with regard to health and disease information, as the extent of their sensitivity should affect the extent of their avoidance behavior.

In addition, the analysis of single screens with testing instruments after each screen could be a fruitful approach to get to more fine-grained insights into attention processes, emotion and affect and their relation to learning outcomes. Combined with this methodological approach, it would be worth to think about a slightly stronger variation of the aversive character of the material by using more aversive material (e.g., personal pictures). In our study, we used exclusively schematic pictures like those that are well-known from educational books. One can assume that the learners categorized the pictures as relatively formal. Another limitation is the test instrument “*per se*.” Our eye tracker was head mounted and had to be re-calibrated after every screen presentation. Participants had to concentrate on sitting still and were subject to interruptions of the learning process during the re-calibrations. These conditions likely hampered the learning process and it is therefore necessary that future studies apply less intrusive methods of recording gaze data.

In summary, the findings of our eye-tracking study show that mixing objective methods and self-reported measurements was a productive approach to obtaining more fine-grained information regarding learning processes. Last but not least, especially data on gaze behavior uncovered mechanisms of the personalization effects that had otherwise been hidden, although the manipulation check told us that the language style was perceived in different ways.

## Ethics Statement

This study was carried out in accordance with the recommendations of Deutsche Forschungsgemeinschaft (German Research Association) with written informed consent from all subjects. All subjects gave written informed consent in accordance with the Declaration of Helsinki. The Bauhaus-Universität Weimar does not hold an own ethical commitee. The Bauhaus-Universität Weimar is member of the German Research Association, at this all researches and employees of the University are committed to act in accordance with the ethical guidelines of the German Research Association. Those are in accordance with the Declaration of Helsinki.

## Author Contributions

SZ: Substantial contributions to conception, acquisition, and interpretation of data for the work; drafting the work. SW: Substantial contributions to acquisition, analysis, and interpretation of data for the work; drafting the work. TK: Substantial contributions to the conception and design of the work and interpretation of data for the work and revising the work critically. SB: Interpretation of data for the work and revising the work critically for important intellectual content and final approval of the version to be published. SZ, SW, TK, and SB: Agreement to be accountable for all aspects of the work in ensuring that questions related to the accuracy or integrity of any part of the work are appropriately investigated and resolved.

## Conflict of Interest Statement

The authors declare that the research was conducted in the absence of any commercial or financial relationships that could be construed as a potential conflict of interest.
